# Investigating the Multidomain Impact of Palliative Care on End-of-Life Patients: A Comprehensive Evaluation

**DOI:** 10.1155/tswj/4203906

**Published:** 2025-01-13

**Authors:** Mohammad O. Abu Hasheesh

**Affiliations:** Basic Nursing Department, Faculty of Nursing, Isra University, Amman, Jordan

**Keywords:** comprehensive evaluation, end-of-life patients, impact, investigating, palliative care domains

## Abstract

**Background:** Palliative care is recognized for its holistic approach in improving the quality of life for patients and their families, focusing on pain relief, symptom management, and addressing emotional, social, and spiritual needs. However, the field is evolving due to increasing demand for these specialized services, emphasizing the need for the ongoing research into palliative care practices.

**Research Purpose:** Is to investigate the multidomain impact of palliative care on end-of-life patients and evaluate their effectiveness on these domains.

**Method:** A quantitative descriptive design was adopted for conducting the current study. Approval obtained from a designated hospital in Amman, Jordan, and official permission to carry out the study. The study's participants consisted primarily of physicians and nurses who were involved in providing care to terminally ill patients. The research tool employed in this study is a standardized palliative care assessment that was used in Australia, with modifications based on the literature review. The validity and reliability of the adapted tool have been ensured through rigorous testing procedures.

**Results:** Findings indicated that the implementation of standardized practical palliative care exhibited an average level across all domains, specifically, the spiritual domain received the highest mean score (1.80), while the structural domain had the lowest (1.69). There were significant differences in palliative care provision across specialized work sites, with radiotherapy and blood disease sites scoring higher (*M* = 2.04 and *M* = 1.87, respectively). Educational levels significantly influenced care perceptions, favoring BSc holders. Age did not significantly affect palliative care provision, probably because of standardized care protocols and sample size. In addition, nurses rated palliative care higher than physicians, likely due to their more direct patient involvement.

**Conclusion:** In light of the results, it is evident that there is a pressing need to consistently evaluate the healthcare services offered to meet the needs of the increasing population of terminally ill patients.

## 1. Introduction

Palliative care is an important public health issue. It is concerned with the suffering, dignity, care needs, and the quality of life of people at the end of their lives [1]. The World Health Organization (WHO) defined palliative care as “an approach that improves the quality of life of patients and their families facing the problem associated with life-threatening illness through the prevention and relief of suffering by means of early identification and impeccable assessment and treatment of pain and other problems, physical, psychosocial, and spiritual” [2].

Palliative care represents a critical component of patient-centered medicine, particularly for individuals nearing the end-of-life [3]. This approach aims to provide comprehensive support that addresses not only physical symptoms but also the psychological, social, spiritual, and cultural dimensions of the patient experience [4]. More specifically, the impact of palliative care on the patient experiencing physical symptoms is definitively associated with reduced suffering, but the nature and role of palliative care in the improvement of the overall quality of patient's life remains under debate in a number of studies [5, 6].

Palliative care emphasizes on multiple domains that enhance the patient's comfort and alleviate suffering [7, 8]. As Jadhav et al. [9] highlight, these domains may include, but are not limited to, physical, psychological, social, and spiritual health-related aspects. Among these domains, the psychological aspect is one of the most important. The previous research has showed that coping strategies, emotional distress, resilience, and social support have provided important predictions of the psychological health of patients requiring palliative care [10, 11]. The social health conditions affect patients and caregivers who participate in palliative care activities. Sultana et al. [6] revealed a relationship between social support, family intervention, and communication on the end of life of people receiving palliative care and established that social aspects have a great influence in this aspect. In addition, the spiritual domain is concerned with fundamental questions of existence, meaning-making, and sources of pain relief for patients at the end of life. In recent investigations of Bovero et al. [12] and Ferrell et al. [13], the importance of these spiritual aspects in the support and well-being of palliative patients is explained. Their findings reveal that spiritual care has tremendous influence on the quality of life of patients in areas of palliative care and stresses that end of life care must incorporate spiritual care to meet patient's needs. Furthermore, cultures dictate patient decision-making in caregiver selection as well as their language of communication and preferred practices at the end of a patient's life, putting into focus, cultural competence in the provision of palliative care [14].

The current evidence highlights the need to evaluate palliative care to enhance its quality and effectiveness for end-of-life patients. To achieve this, there is a need to understand the complexity of the palliative care domains so as to develop the best solution that will address the needs of the clients receiving the care. Thus, this study intended to investigate the effects of palliative care domains on the quality of the care delivered to patients at their final stage of life with a view of comparing the gaps and promoting well-being.

### 1.1. Research Question

The emphasis of this study is to examine the impact of domains of physical, psychological, social, spiritual, cultural, and structural effects on the quality of palliative care that patients receive near their end of life from the healthcare practitioners. This study aims to address the following specific research questions:

1. What is the level of standard palliative care delivered for terminally ill patients?
2. Is there a difference in the level of standardized palliative care provided to the patients and the sociodemographic characteristics of the studied sample?

### 1.2. Significance of the Study

Palliative care plays a pivotal role in enhancing the quality of life for individuals facing terminal illnesses, yet understanding the nuanced domains of palliative nursing care remains critical for optimizing patient outcomes. By addressing the holistic needs of end-of-life patients, this study contributes to advancing palliative care practices, enhanced patient outcomes, and improved quality of life during the final stages of illness.

## 2. Methods

### 2.1. Design

A quantitative descriptive design was employed to thoroughly investigate and evaluate the quality of palliative care provided to terminally ill patients, focusing on various domains affecting end-of-life care. This type of design is common in research that uses numerical data to describe the characteristics of a population or phenomenon being studied without manipulating variables [15].

### 2.2. Setting

The study took place at a public hospital in Amman, Jordan, selected to assess and explore the impact of palliative nursing care domains on the quality of end-of-life care for patients receiving palliative care. The choice is based on the accessibility, catering extensively to the population of Amman city, particularly oncology patients.

### 2.3. Population and Sampling

The study population participated in this study was the entire class of nurses and physicians as healthcare professionals providing a palliative care for terminally ill patients in various healthcare settings. A total of 93 participants were included in the study, comprising 41 physicians and 52 nurses. They were recruited from five departments that handle terminally ill patients, namely, intermediate, internal medicine, pediatrics, radiotherapy, and blood diseases. Detailed characteristics of the participants are presented in Table 1.

### 2.4. Data Collection Procedure

Prior to commencing data collection, approval was sought and obtained from the Institutional Review Board (IRB) at Isra University. The approval process ensured that the study adheres to ethical standards and protects the rights and welfare of the participants involved. Approval was also obtained from the hospital administration where the study was conducted. They reviewed the study protocol and provided authorization for the researcher to access relevant healthcare providers for data collection. In addition, approval was secured from the head departments. They endorsed the study based on its relevance to clinical practice and potential benefits to improve palliative care services for end-of-life patients. Participants completed structured questionnaires designed to assess their perceptions of various domains of palliative care.

### 2.5. The Research Instrument

The level of standardized palliative care was investigated using the Australian standardized palliative care model, which is widely regarded for its parsimony and empirical validation. The model was the fifth revised edition of the Standards for Palliative Care Services; it organizes the standards into six domains based upon the needs of the patient and family. These quality domains are underpinned by core values, and principles are focused on the needs of patients, families, staff, volunteers, and the wider community. It is widely used in palliative care settings to ensure that the care provided aligns with holistic principles, which focus on addressing the physical, emotional, social, and spiritual needs of patients [16]. The validity and reliability have been demonstrated by previous studies [17]. The original tool was modified to address the cultural and linguistic needs of the target population. Modifications were made according to expert consultation, including simplifying language for clarity and understanding, as well as adding questions to meet spiritual needs of participants. Pilot testing of the study tool was conducted to assess internal consistency reliability, with Cronbach's alpha of +0.82 for the total of 106 items.

The study instrument used in the current study evaluates the multidomain impact of palliative care, covering the physical, psychological, social, spiritual, cultural, and structural domains that align with the diverse needs of patients and their families. It also incorporates demographic questions regarding age, gender, level of education, occupation, years of experience, and departmental affiliation.

The study instrument comprised of 106 items across six domains, each scored on a 4-point Likert scale (0 = *not done*, 1 = *sometimes done*, 2 = *frequently done*, and 3 = *always done*). Participants were instructed to assess their responses to items within the scale. The total score was computed statistically using predefined ranges (0–0.75 for not done, 0.76–1.5 for sometimes done, 1.51–2.25 for frequently done, and 2.26–3 for always done), which were transformed to generate an overall practice level score for different departments involved in the study.

### 2.6. Data Analysis

Quantitative data collected from questionnaires were analyzed using statistical software (SPSS, version 28). Participant characteristics including age, educational level, experience, profession type, gender, and department/workplace were summarized using frequencies and percentages. The palliative care domains were presented in terms of means, standard deviations, and percentages. In addition, *F*-tests and *T*-tests were employed to examine the relationships between various palliative care domains and the sociodemographic characteristics of the participants.

### 2.7. Ethical Considerations

The study protocol received approval from the Research Ethics Committee at Isra University (Jordan), with the ethics approval number: “SREC/23/05/079. This was followed by obtaining approval from the targeted hospital. Participants were informed about the purpose, benefits, risks, and voluntary participation. Participating nurses and physicians signed consent forms. The study was conducted in accordance with the Declaration of Helsinki to safeguard participants' rights and ensure the confidentiality of their personal information.

## 3. Results

As presented in Table 1, the sociodemographic and personal characteristics of the study sample reveal the largest age group observed is the 41–50 years category, which comprises 40 participants, representing 43.5% of the total sample. This suggests a strong representation of middle-aged individuals in the study. Regarding educational level, the majority (91.9%) held a Baccalaureate degree. Concerning years of experience, 48.4% had less than 10 years, 35.2% ranged between 11 and 20 years, and 16.5% had over 20 years of experience. In terms of gender distribution, 64.1% were male and 35.9% were female. Professionally, 55.9% were nurses and 44.1% were physicians. In terms of departmental affiliation, the largest representation came from the radiotherapy department with 51.6% of the sample. This was followed by the blood disease and pediatric departments, both at 12.9%, the intermediate department at 12.9%, and finally the internal department at 7.5%.

The data summarized in Table 2 present the descriptive statistics for different domains of palliative care. The spiritual domain had the highest mean score ((*M* = 1.80, SD = 0.74, 60%), while the structural domain exhibited the lowest mean score (*M* = 1.69, SD = 0.77, 56.3%). The levels of care were similar for the physical and psychological domains, both at 59.8%. Following were the social and cultural domains, with percentages of 59% and 57.6%, respectively. Basically, several strategies could be considered focusing on structural domain and emphasizing the spiritual domain. Overall, Table 2 indicates that palliative care provided to terminally ill patients was frequently administered across all domains (mean = 1.76, within the range of 1.51–2.25).

The mean scores and standard deviations across various work sites of palliative care (intermediate, internal, pediatric, radiotherapy, and blood disease) and for the total domain as shown in Table 3, suggesting potential differences in the provision palliative of care experienced by healthcare providers in different specialized contexts. Significant statistical differences exist across different specialized work sites, indicating that the application of entire domains of palliative care and work sites significantly related to favor radiotherapy (*M* = 2.04), followed by blood disease work sites (*M* = 1.87). Overall, Table 3 indicates a potential variation in palliative care delivery based on work sites, with an F-value of 8.60 and a *p* value of < 0.001, further supporting these differences.

In examining the level of palliative care provided among educational levels of the participants, as detailed in Table 4, a statistically significant difference among the educational levels being compared was found in favor of BSc holders (*p* value: 0.049; *F-*value: 3.12), suggesting that educational levels influence how end-of-life patients perceive or experience various aspects of palliative care. Overall, this is crucial for understanding how educational attainment might influence perceptions of palliative care quality, informing strategies for improving care provision across different educational backgrounds.

The data on palliative care provision across various age groups revealed an average provision score of (*M* = 1.8) and a standard deviation of (SD = 0.66) across all age categories. The *F*-value of 0.35 and associated *p* value of 0.78 indicate that there is no statistically significant difference in palliative care provision among different age groups. These findings could be due to the homogeneity of care and standardized protocols provided by health care providers, as well as a small sample size of the study, see Table 5.

The p value for the comparison between nurses and physicians in Table 6 was found to be 0.001, suggesting a significant difference between nurses (*M* = 2.0) and physicians (*M* = 1.7), particularly nurses rating palliative care provision significantly higher (*t* = 3.34, *p*=0.001). This disparity could be attributed to nurses' more involvement in direct patient care and holistic support, fostering a deeper appreciation for the quality of care provided. In addition, gender did not significantly impact perceptions, as both male (*M* = 1.72) and female (*M* = 1.79) respondents rated palliative care provision care similarly (*t* = 0.49, *p*=0.62 for males; *t* = 0.47, *p*=0.63 for females). These findings highlight the nuanced differences in how healthcare professionals perceive palliative care, emphasizing the crucial role of direct patient interaction and professional roles in shaping these perceptions.

## 4. Discussion

The current study examines the importance of palliative care domains; however, it should be noted that these results are specific to the local context of nurses and physicians in a public hospital in Amman, Jordan, and they may vary in other contexts due to differences in organizational and cultural structures. The spiritual and psychological aspects of care received high importance ratings of 60% and 59.8%, respectively, and at the same time, the structural domain obtained a lower overall importance rating. This finding suggests that despite patients in this category receiving significant support in the area of spiritual and psychological care, there may be significant gaps in classic structural administrative support for palliative care services. A number of research studies also bear the fact that spiritual and psychological care in the palliative settings is appreciated. Kozlov et al. [18] revealed that spiritual support is an area where palliative care services often excel, which correlates with the higher results of work in the sphere of spirituality. The study found out that the value of spirituality was embraced since patients were satisfied with the spiritual care they received during the terminal illness. Similarly, Abramson [19] has established that the psychological aspect plays an important role in the delivery of palliative care as patients' satisfaction toward comprehensive psychological consultations, and treatments were very high. His results are consistent with the finding of this study, that the use of those palliative care services boost the level of satisfaction of the patient and improve the quality of care when a specialized psychological support service is integrated in the service. On the other hand, the observed low score in the structural domain in the present study synchronizes with other related studies identifying systematic barriers to palliative care. Moreland et al. [20], pointed out that although changes in the palliative care practice have been observed, many healthcare institutions limited access to basic infrastructure and administrative support which pose a significant threat for quality provision of the services. Potential environmental constraints mapped out by their research include inadequate structural support that compromises the delivery of optimal palliative care, measures of resources, and the care team coordination. Moreover, Hickman et al. [21] noted that there are structural problems, including staff shortages and the lack of well-coordinated pathways, which result in less positive patients' experience. This is in harmony with the current study's finding indicating a low structural domain score, thereby implying the development of infrastructure and the administrative processes to enhance the quality of palliative care services.

The present research also found statistically significant differences in mean scores in the work site of palliative care provision, specially, in the Radiotherapy and the Blood Disease departments, where the mean scores were 2.04 and 1.87, respectively. On this basis, there is an implication that the quality of care may be determined by features of work sites. The research conducted in palliative care workplaces also confirms findings about relative differences in palliative care quality across different specialty sectors. For instance, in a study by Bazargan and Bazargan-Hejazi [22], concerns were raised regarding the quality of palliative care that patients received in specialized facilities as compared to other general nononcology wards. They also highlight how medical specialty can influence the provision and reception of palliative care, which aligns well with the variation observed in the current study between radiotherapy and hematology and other departments. Furthermore, the systematic review by Gardiner et al. [23] synthesized data from multiple studies and suggested that even though demographic characteristics of patients and their medical conditions influence care outcomes, institutional factors and care processes can reduce the effects of medical specialization on the perceived quality of care.

A positive correlation between the education level and perception of the palliative care domains among the end-of-life patients was anticipated. Specifically, the analysis reveals a statistically significant difference in favor of BSc holders (*p* value: 0.049; *F*-value: 3.12). This is an implication that general knowledge or prior exposure influences how healthcare providers interpret or even encounter different aspects of palliative care. The previous research supports the findings of the current study regarding educational impacts on healthcare perception. Sourady et al. [24] explained that the use of the palliative care services depends on the educational levels of the people, and the highly educated persons were more conscious of the palliative care service than the less educated persons. Similarly, Wong et al. [25] revealed that healthcare providers who had more education background were more likely to perceive more positive aspects in giving palliative care. On the contrary, Kinley et al. [26] indicated a null correlation between education levels and levels of satisfaction with the palliative care, implying that other factors may tend to outweigh education in such settings. In addition, Elmelegy et al. [27] revealed that although education affects patient's knowledge of available treatment practices, it has little impact on actual perceptions of palliative care dimensions.

The current study revealed that there was no significant increase or decrease in the delivery of palliative care with the age of healthcare givers. The research literature reviewed in this paper shows conflicting evidence of age differences in palliative care. For instance, as current studies agree that age was not a potent factor in care outcomes, Tanuseputro et al. [28] also noted that the provision of palliative care was similar across age cohorts. This brings about variations in the way healthcare providers administer palliative care. Andersson et al. [29] reported that the quality of palliative care did not vary with age and supported the idea that the needs of the patients are not limited to age factors. On the other hand, some authors describe the following gaps to be quite substantial by the age factor. A similar example is a study by Dyer et al. [30], who indicated that young patients were not fully evaluated and treated as clients, most likely because of clinician prejudice. This view counters our findings and implies that age affects the quality of palliative care delivery in certain circumstances. Such discrepancies in the current study might indicate that currently healthcare providers are very competent and committed to provide equal healthcare services regardless of the age of patients as provided by a review by Gazaway et al. [31]. Palliative care appears to be a specialized area in which a widespread need for standardization to develop across the healthcare system.

Regarding the organizational conflict about the quality of patient care between physicians and nurses, the current study results showed that nurses rated the management's provision of palliative care significantly higher (*M* = 2.0) compared to the physicians' lower score (*M* = 1.7), *t* = 3.34, *p*=0.001. The reason for this gap might be found in the fact that nurses are directly involved in patient care and focus on comprehensive care of the patient and the patient's family. Similar to Hagan et al. [32] and Harden et al. [33], they noted a great value of the continuous presence of staff in enriching the quality of the palliative care since the close contact allows building long-term relationships with the clients to better understand their needs. Furthermore, comparing investigations executed by physicians and nurses stated differences between the approaches of the two professions, one can observe contradicting opinions in some cases. Yi [34] observed that physicians may have a self-generated bias because of the clinical focus and training and rate the quality of palliative care higher because of their target of medicoscientific values. This shows how team members need to engage in a conversation across different professions in order to solve patient care dilemmas and to advance knowledge of what each profession can offer. In addition, the findings can help to expand the current discussion about the role of comprehensive care in the development of palliative services. The elevated rate of satisfaction by nurses may be because of more extensive perception of other aspects of patient care encompassing emotional, psychological, and social aspects as noted by Carter et al. [35]. That is why there is a growing interest in training that focuses on the interprofessional and different approaches to work in palliative care.

The current study demonstrated that no significant differences existed in the perceived provision of palliative care with both male participants (*M* = 1.72) and female participants (*M* = 1.79) (*t* = 0.49, *p*=0.62). This insight corresponds with the research of Sourady et al. [24], where it was stated that the attitudes of healthcare professionals differ slightly in males and females. Thus, it might be that factors other than gender, namely, professional role and direct patient contact that have an even stronger effect. It is dissimilar to those other works that have focused on gender as an important aspect of perceptions on palliative care [36, 37]. Thus, the current study asserts that the provision of palliative care services is affected by the contextual and professional factors rather than the gender factor alone.

Although the study provided valuable insights into the experience of nurses and physicians in a public hospital in Amman, Jordan, there are limitations that should be considered. One limitation is that the sample was drawn from one geographic and institutional context. Therefore, caution should be exercised when attempting to generalize the results to other countries or institutions, and additional studies in diverse contexts are needed to enhance generalizability.

### 4.1. Implications

The findings from this study are valuable to practice and policy-building, in particular, healthcare practitioners and institutions, and policymakers regarding the gaps where palliative care can be enhanced. Developing knowledge of the various aspects of palliative care that that can enhance the quality of life and provide better care for patients in their final stages.

### 4.2. Recommendations

The following recommendations are put forth: The comprehensive training for personnel in delivering standardized palliative care, integration of palliative care courses into the nursing curriculum, and the promotion of workshops and learning activities to enhance healthcare professionals' skills as part of staff development and ongoing education. These initiatives are imperative for enhancing the overall quality of patients receiving palliative and end-of-life care.

## Figures and Tables

**Table 1:** Distribution of the participants according to their sociodemographic and personal characteristics.

Variable	Category	Frequency	Percent (%)
Age	21–30	18	19.6
	31–40	27	29.3
	41–50	40	43.5
	51–60	8	7.6
	Total	93	100

Educational level	Baccalaureate	79	91.9
	Higher education	9	2.3
	Diploma	5	5.8
	Total	93	100

Experience	Less than 10 years	44	48.4
	11–20 years	32	35.2
	21–30 years	10	11
	More than 30 years	7	5.5
	Total	93	100

Gender	Male	59	64.1
	Female	34	35.9
	Total	93	100

Profession	Nurse	52	55.9
	Physician	41	44.1
	Total	93	100

Department	Intermediate	12	12.9
	Internal	7	7.5
	Pediatric	12	12.9
	Radiotherapy	48	51.6
	Blood disease	14	15
	Total	93	100

**Table 2:** Means, standard deviations, and percentage for the level of palliative care provided to terminally ill patients.

Domains of palliative care	Physical	Psychological	Social	Spiritual	Cultural	Structural	All domain
Mean	1.79	1.79	1.77	1.80	1.73	1.69	1.76
Standard deviation	0.66	0.66	0.77	0.74	0.84	0.77	0.65
Percent (%)	59.8	59.8	59	60	57.6	56.3	58.6

*Note:* ∗0–0.75 not done; ∗0.76–1.5 sometimes done; ∗1.51–2.25 frequently done; ∗2.26–3 always done; *N* = 93.

**Table 3:** Analysis of provision of entire domains of palliative care by work sites: *F*-test results.

Palliative care provision	Variables	Categories	No. of sample	*M*	SD	*F* value	*p* value
Palliative care (whole domain)	Work sites	Intermediate	12	1.07	0.71	8.60	< 0.001
		Internal	7	1.86	0.41		
		Pediatric	12	1.57	0.64		
		Radiotherapy	48	2.04	0.54		
		Blood disease	14	1.87	0.40		
		Total	93	1.76	0.65		

*Note:* The *p* value (< 0.001) in Table 3 indicates that the differences in the provision of palliative care across the various work sites (intermediate, internal, pediatric, radiotherapy, and blood diseases) are statistically significant.

**Table 4:** Analysis of whole domains of palliative care provision by educational levels: *F*-test results.

Palliative care provision	Variables	Categories	No. of sample	*M*	S D	*F* value	*p* value
Palliative care (whole domain)	Educational levels	BSc	79	1.78	0.64	3.12	0.049
		Higher education	2	1.09	0.45		
		Diploma	12	1.19	0.28		
		Total	93	1.73	0.64		

**Table 5:** Analysis of whole domains of palliative care provision by age: *F*-test results.

Palliative care provision	Variables	Categories	No. of sample	*M*	SD	*F* value	*p* value
Palliative care (whole domain)	Age	21–30 years	27	1.7	0.71		
		31–40 years	40	1.8	0.60		
		More than 40 years	8	1.9	0.71		
		Total	93	1.8	0.66	0.35	0.78

**Table 6:** Analysis of whole domains of palliative care provision by profession and gender: *t*-test results.

Palliative care provision	Variables	Category	No. of sample	*M*	SD	*T* value	*p* value
Palliative care (whole domain)	Profession	Nurses	52	2.0	0.50	3.47	0.001
		Physicians	41	1.7	0.69	3.34	0.001

Palliative care (whole domain)	Gender	Male	59	1.72	0.62	0.49	0.62
		Female	33	1.79	0.71	0.47	0.63

## Data Availability

The data that support the findings of this study are available on request from the corresponding author. The data are not publicly available due to privacy or ethical restrictions.
